# Latent Profile Analysis of Moral Foundations: Emotional and Decisional Forgiveness Approaches to Models of Morality

**DOI:** 10.1002/ijop.70009

**Published:** 2025-02-03

**Authors:** Sebastian Binyamin Skalski‐Bednarz, Loren L. Toussaint, Anna Kwiatkowska, Adrianna Mendrek

**Affiliations:** ^1^ Faculty of Philosophy and Education Katholische Universität Eichstätt–Ingolstadt Eichstätt Germany; ^2^ Institute of Psychology Humanitas University Sosnowiec Poland; ^3^ Department of Psychology Luther College Decorah Iowa USA; ^4^ Institute of Psychology Polish Academy of Sciences Warsaw Poland; ^5^ Department of Psychology Bishop's University Sherbrooke Quebec Canada

**Keywords:** decisional forgiveness, emotional forgiveness, latent profile analysis, moral foundations, morality

## Abstract

This study delves into the intricate relationship between morality and episodic forgiveness (i.e. emotional and decisional), guided by moral foundations theory. Survey data were collected from 927 English‐speaking Canadians, aged 18–57, using the Moral Foundations Questionnaire, Decision to Forgive Scale, and Emotional Forgiveness Scale. Employing latent profile analysis, the research revealed three distinct moral foundation profiles—high moralists, individuators, and neutrals—each linked to different levels of decisional and emotional forgiveness. Further analysis using MANOVA and follow‐up ANOVAs indicated that the high moralist group exhibited higher scores in both forgiveness dimensions compared to the individuator and neutral groups, whereas the individuator group reported higher emotional forgiveness than the neutrals. These findings illuminate the significance of moral development in forgiving and underscore the utility of moral profiling based on moral foundations theory in predicting episodic forgiveness.

## 1 Introduction

Forgiveness inherently occurs within the context of a transgression (McCullough and Hoyt 2002). Perspectives on transgressions and their conceptions are closely tied to individuals' moral outlooks, influencing the likelihood of granting forgiveness (Bassett et al. 2018). Forgiveness, grounded in various philosophical, social, religious, and spiritual perspectives, represents not only a valuable response to wrongdoing but also significantly contributes to personal flourishing—a state associated with optimal well‐being in positive psychology (Agenor, Conner, and Aroian 2017; Keyes 2002; Seligman 2011). This pursuit of flourishing extends beyond survival, encompassing engagement, meaning, and accomplishment (Diener et al. 2010), thereby influencing resilience amidst adversity (VanderWeele 2017). Philosophical traditions, particularly Aristotle's virtue ethics, emphasise virtues in flourishing, with forgiveness emerging as a critical moral virtue for well‐being (Aristotle 1925; Enright and Coyle 1998). Enright, Freedman, and Rique (1998) define episodic forgiveness as a deliberate choice to release feelings of vengeance and bitterness towards someone who caused harm, aiming instead for benevolence, empathy, and goodwill in response. Worthington (2020) further describes episodic forgiveness as an intentional, voluntary process involving letting go of negative emotions towards the offender, fostering empathy and understanding, and ultimately facilitating healing and inner peace. This conceptualisation of episodic forgiveness encompasses two dimensions. Decisional forgiveness refers to the cognitive decision to relinquish resentment and embrace a more positive and compassionate attitude towards the offender (Lichtenfeld et al. 2015). In contrast, emotional forgiveness entails internally releasing negative emotions linked to the transgression, reducing feelings of anger, bitterness, and vengefulness (Worthington et al. 2007). Integrating these aspects enables individuals to move beyond hurt without condoning the behaviour or forgetting its consequences (Toussaint et al. 2020).

Enright's (2019) Process Model provides a step‐by‐step process, depicting phases such as uncovering, decision, work, and deepening, and Worthington's (2019) REACH Forgiveness model, emphasising recall, empathising, altruistic gift, committing, and holding onto forgiveness, illustrates episodic forgiveness as a gradual process involving understanding, acceptance, and the release of negative emotions. The research underscores the myriad benefits of episodic forgiveness for mental health, reducing stress, depression, and anxiety, and enhancing interpersonal relationships and life satisfaction (Lee and Enright 2019; Rasmussen et al. 2019; Skalski et al. 2022; Toussaint et al. 2023). Nevertheless, the complexity of forgiveness requires further exploration of individual influences in episodic forgiveness. Understanding its intricacies can aid in developing interventions and therapeutic approaches promoting forgiveness and emotional healing following transgressions.

## 2 Morality and Forgiveness

Philosophers hold varying perspectives on forgiveness. Murphy (1988) describes it as a discretionary moral action influenced by emotional responses and the need for self‐respect. Coleman (2003) sees forgiveness as a form of moral repair, aiding in the restoration of social harmony and relationships. Griswold (2007) and Nussbaum (2016) emphasise its potential for personal growth and moral development. Some philosophers extol forgiveness as virtuous and closely tied to divine will or moral goodness (Garcia 2011; Griswold 2007), while others view it as a human action, offering an objective interpretation and leaving ethical assessments open for each instance of forgiveness (Hieronymi 2001; Kolnai 1974; Russell 2016; Zaibert 2009). This divergence in philosophical views sparks significant debates regarding the ethical essence of forgiveness, wielding substantial influence over the forgiveness process due to its inherent moral ambiguity (Ingersoll‐Dayton and Krause 2005; Lamb and Murphy 2002). Qualitative studies indicate individuals' reluctance to forgive unless amends or apologies are made (Ingersoll‐Dayton and Krause 2005, 279). Similarly, Carpenter, Carlisle, and Tsang (2014) highlighted in quantitative research that conciliatory behaviour influences the perceived moral appropriateness of forgiveness. However, further research is required to enhance our understanding of the intricate relationship between morality and forgiveness.

Among the various theories explaining differences in moral values, moral foundations theory (Graham et al. 2013) has been particularly influential, enhancing our comprehension of moral decision‐making. While the above literature review primarily draws from philosophical perspectives on the moral justifiability of forgiveness due to their prevalence in the field, this work aims to explore how moral principles may promote episodic forgiveness, which can be examined across many other disciplines.

## 3 Moral Foundations

Episodic forgiveness is influenced by multiple factors, including personal experiences and contextual elements. Recent research, exemplified by Benard et al. (2022), underscores the significant impact of morality on individuals' inclination to forgive transgressions. Exploring individual differences within these moral frameworks provides crucial insights into the diverse ethical perspectives that form the basis of forgiveness. According to moral foundations theory (Graham et al. 2013), five inherent moral foundations shape individuals' moral reasoning, influencing their attitudes, beliefs, and behaviours across diverse social and political landscapes: care/harm, emphasising harm avoidance and aiding those in need; fairness/cheating, focused on reciprocity and condemning exploitative behaviour lacking reciprocation; loyalty/betrayal, valuing group allegiance while condemning betrayal; authority/subversion, involving respect for hierarchy and traditions; and sanctity/degradation, highlighting reverence for the body and aversion to contamination. These foundations are classified as individualising foundations—care/harm and fairness/cheating, which prioritise individual rights and welfare—and binding foundations—loyalty/betrayal, authority/subversion, and sanctity/degradation, which emphasise group cohesion and social order. Together, they help delineate interpersonal interactions and group dynamics within larger institutions (Haidt 2012). Despite their presumed cross‐cultural universality, variations in these foundations may arise due to cultural, social, and individual factors (see Hofstede 2001).

While the moral foundations theory remains a widely accepted and influential framework for understanding morality, it is not without its critiques. Some suggest that it may not fully encompass all significant moral elements, particularly in diverse cultural contexts, and that it may place too much emphasis on cognitive processes at the expense of emotional components (see Graham et al. 2013). Nevertheless, it continues to be a gold standard in the field, offering a comprehensive approach to studying moral reasoning and ethical behaviour across different societies.

The care/harm and fairness/cheating morals are often considered “default” morality in Western societies and are most frequently employed (Leidner and Castano 2012). However, other moral foundations can become more influential in certain situations, potentially altering behaviour interpretations and reducing the emphasis on harm avoidance (Hare 1981). To maintain their moral self‐image, individuals may shift between moral foundations, sometimes unconsciously, aligning with Haidt and Bjorklund's (2008) concept of moral intuitions—automatic moral judgements based on underlying moral foundations.

Definitions of episodic forgiveness often highlight the moral foundations of care/harm and fairness/cheating, framing forgiveness as a response to perceived unfair or hurtful transgressions (Enright and Coyle 1998; VandenBos 2007; Worthington 2020). However, Lindsey's (2014) study introduces the moral foundation of sanctity/degradation as another significant aspect of episodic forgiveness. Lindsey suggests that forgiving moral transgressions related to sanctity can offer similar physical and mental health benefits (Lawler‐Row and Piferi 2006), relational advantages (Hook, Worthington, and Utsey 2009), and a sense of empowerment for victims as forgiveness connected to harm and fairness. This underscores the multifaceted nature of moral considerations in the context of episodic forgiveness.

Furthermore, Bassett et al. (2018) indicated that moral foundations influence forgiveness through changes in empathy, grace orientation, and narcissism. These authors also suggested that an individual's moral standards might affect his or her perception of the seriousness of an offence, with various studies demonstrating the impact of offence severity on the forgiveness process (Dolan 2003; Leidner and Castano 2012).

Based on the literature review, the evolution of moral foundations significantly shapes individuals' belief systems, profoundly impacting their motivation for behaviour, including the act of forgiveness. For example, individuals who prioritiseindividualising foundations over binding foundations may exhibit greater forgiveness due to heightened empathy and sensitivity to suffering (Clark et al. 2017; Nilsson et al. 2016; Shepherd et al. 2018). However, a prevalent challenge in this area lies in oversimplifying the breadth of human morality by categorising individuals based on their endorsement of individual‐focused versus group‐focused moral concerns, such as ingroup loyalty (e.g. Graham, Haidt, and Nosek 2009). Extensive research indicates that moral foundation profiles exhibit a more nuanced and diverse structure than this binary classification implies (e.g. Haidt, Graham, and Joseph 2009; Milojev et al. 2014; Weber and Federico 2013). For instance, Skalski‐Bednarz et al. (2023) conducted multiple mediator model analyses, proposing that the link between religion and environmental concern might be shaped by complex moral profiles interacting and collectively contributing to the advancement of environmental consciousness. Conversely, in a nationally representative New Zealand sample, Milojev et al. (2014) employed latent profile analysis (LPA) to identify four profiles—*high moralists* (high scores across all five moral foundations), *individuators* (high individualising foundation scores but lower binding foundation scores), *moderates* (mid‐level scores across all foundations), and *neutrals* (low scores across all foundations)—revealing distinctive combinations of moral foundations within these profiles. Additionally, Greenway et al. (2019) observed significant differences across these four profiles in Americans, associated with variations in generosity, specific components of empathy, religiousness, and political ideology. Similarly, using mixed‐mode latent class analysis among US undergraduate students, Weber and Federico (2013) categorised stances on social issues like crime, environment, immigration, and abortion, revealing six distinct representations of morality. Considering these findings, it seems that moral foundation profiles may similarly advance prosocial studies, such as investigations into forgiveness.

## 4 Present Study

This study aims to enhance our comprehension of the associations between moral foundations and episodic forgiveness (i.e. emotional and decisional). Building upon the multifaceted nature of moral foundations evidenced in prior studies, we hypothesize that latent profiles delineating distinctive moral intuitions—such as high moralists, individuators, moderates, and neutrals—to manifest using the Moral Foundations Questionnaire (MFQ), consistent with previously identified profiles (Greenway et al. 2019; Milojev et al. 2014). However, in light of the lack of empirical evidence regarding how measures of episodic forgiveness differ across distinct profiles (beyond the general assumption of positive links between morality and forgiveness) (Garcia 2011; Griswold 2007; Lindsey 2014), we leave open‐ended the relationship between these profiles and decisional as well as emotional forgiveness.

## 5 Materials and Methods

The investigation was conducted in the fall of 2023 using the online Prolific survey platform after receiving approval from the university's ethics committee. A total of 927 English‐speaking Canadians participated, ranging in age from 18 to 67 years (*M* = 45.3, SD = 9.87), with women constituting 51% of the sample. All individuals self‐identified as cisgender. Participation in the study did not necessitate meeting specific recruitment criteria; each participant provided informed consent before engaging. Among the participants, 78% resided in urban areas, while 22% lived in rural regions. Marital status varied, with 47% of respondents being married, 29% single, 16% divorced, and 8% widowed. A majority (61%) reported a Christian religious affiliation, while the remaining 39% identified as agnostic or non‐believers. The survey included questionnaires assessing moral foundations and episodic forgiveness, with the MFQ administered first. Participants were then asked to recall instances of harm they had experienced from another person in the past five years, without controlling for the type and severity of these transgressions. The survey took approximately 20 min to complete, and participants were compensated 8 Canadian dollars (CAD) for their participation.

### 5.1 Measures

#### 5.1.1 Moral Foundations

The MFQ by Graham et al. (2011) was used to evaluate moral foundations relevant to ethical decision‐making. This questionnaire, comprising 30 items, assesses the five dimensions of morality posited by moral foundations theory (Graham et al. 2013): *care/harm* (*α* = 0.78, all alphas are reported from the present data; e.g. “Compassion for those who are suffering is the most crucial virtue”), *fairness/cheating* (*α* = 0.80; e.g. “When the government makes laws, the number one principle should be ensuring that everyone is treated fairly”), *loyalty/betrayal* (*α* = 0.84; e.g. “It is more important to be a team player than to express oneself”), *authority/subversion* (*α* = 0.86; e.g. “Respect for authority is something all children need to learn”), and *sanctity/degradation* (*α* = 0.91; e.g. “I would call some acts wrong because they are unnatural”). The care/harm and fairness/cheating dimensions represent individualising foundations, while the loyalty/betrayal, authority/subversion, and sanctity/degradation dimensions represent binding foundations. The MFQ comprises two sections. In the initial segment, participants rated the relevance of various considerations to their moral decision‐making on a six‐point Likert‐type scale 1 (*Not very relevant*) to 6 (*Extremely relevant*) (e.g. “Whether or not someone cared for someone weak or vulnerable”, aligning with the care/harm scale). In the subsequent section, participants responded to statements using a six‐point Likert‐type scale, ranging from 1 (*Strongly disagree*) to 6 (*Strongly agree*) (e.g. “People should be loyal to their family members, even when they have done something wrong”, reflecting loyalty/betrayal).

#### 5.1.2 Episodic Forgiveness

The Decision to Forgive Scale (DTFS) developed by Davis et al. (2015) was utilised to assess decisional forgiveness, defined as “the cognitive process of letting go of resentment, bitterness, and the desire for vengeance” (DiBlasio 1998, 78). Decisional forgiveness encompasses a cognitive aspect that alters one's intentions concerning behaviour towards an offender, notably addressing motivations related to revenge and avoidance (Exline et al. 2003). Comprising eight statements forming a single factor (*α* = 0.90), the DTFS requires participants to rate five items on a five‐point Likert scale of 1 (*Extremely uncharacteristic*) to 5 (*Extremely characteristic*). Sample items include: “I have decided to forgive him or her”; “I made a commitment to forgive him or her”.

The Emotional Forgiveness Scale (EFS) designed by Hook et al. (2012) was employed to assess emotional tranquillity and forgiveness concerning a specific offence the participant chose. The EFS comprises eight items that gauge the presence of positive and prosocial sentiments towards the perpetrator and the reduction of negative emotions towards the offender. These items are categorised into two factors: presence of positive emotion (*α* = 0.81) and reduction of negative emotion (*α* = 0.84). Since these domains are strongly inter‐correlated, we used the total EFS score. In the EFS, participants are tasked with rating each statement on a five‐point Likert‐type scale of 1 (*Extremely uncharacteristic*) to 5 (*Extremely characteristic*). Sample items include: “I feel sympathy toward him or her”; “I resent what he or she did to me”.

### 5.2 Data Analysis

In the initial step, analyses such as initial data screening, descriptive statistics, alpha coefficients, and bivariate and biserial correlations were run (see Table 1). After that, LPA was explored to discern consistent profiles within the sample based on their moral foundation domain scores (Marsh et al. 2009). This methodology allows for the inference of class membership concerning interactions within the moral foundation domains: care/harm, fairness/cheating, loyalty/betrayal, authority/subversion, and sanctity/degradation. Moreover, based on the collected data, these classes can examine individual differences in episodic forgiveness. We used the following criteria to assess the best model fit: the bootstrap likelihood ratio test (BLRT; higher value = better fit), Bayesian Information Criteria (BIC; smaller value = better fit), sample size‐adjusted BIC (SSABIC; smaller value = better fit), entropy values (higher values = better fit), and average latent class probabilities for each profile solution (for further detail, see Gustafsson et al. 2016; Rumbold et al. 2021). Five hundred random start values were employed for each model to ensure robustness, with the final selection retaining the 50 best among them. Additionally, 1500 random start values were utilised to circumvent potential local maxima issues (cf. Gustafsson et al. 2016). The subsequent phase involved a multivariate analysis of variance (MANOVA) to investigate differences among moral foundation profiles regarding episodic forgiveness. Since sex and age were not significantly correlated with forgiveness, the MANOVA did not include them as covariates. For post hoc comparisons, we utilised the sequential Hochberg (1988) method, a “step‐up” approach that serves as a more potent alternative to the commonly used Bonferroni procedure. This method involves a series of steps in the correction, with each step depending on the result of the preceding one. All analyses were conducted in Jamovi software.

**TABLE 1:** Descriptive statistics and correlations (*N* = 927).

	*M* (*SD*)	1.	2.	3.	4.	5.	6.	7.
1. Care/harm	4.63 (0.58)	—						
2. Fairness/cheating	4.35 (0.64)	0.58***						
3. Loyalty/betrayal	3.4 (0.80)	0.23***	0.26***					
4. Authority/subversion	3.17 (0.85)	0.16***	0.18***	0.66***				
5. Sanctity/degradation	3.57 (0.96)	0.28***	0.26***	0.62***	0.67***			
6. Decisional forgiveness	2.79 (0.74)	0.19***	0.16***	0.18***	0.18***	0.24***		
7. Emotional forgiveness	3.54 (1.05)	0.14***	0.07*	0.15***	0.14***	0.16***	0.52***	
Age	45.3 (9.87)	0.10**	0.09**	−0.02	0.05	0.03	0.04	0.02
Sex (0 = male, 1 = female)		0.12***	0.14***	−0.10**	−0.09**	−0.02	−0.03	−0.01

** *p* < 0.01.

*** *p* < 0.001.

## 6 Results

The correlation analysis revealed significant positive associations between each moral foundation and the domains of episodic forgiveness. Additionally, the analysis included their associations with age and sex (where 0 represents males and 1 represents females). Specifically, care/harm exhibited positive correlations with fairness/cheating (*p* < 0.001), loyalty/betrayal (*p* < 0.001), authority/subversion (*p* < 0.001), sanctity/degradation (*p* < 0.001), decisional forgiveness (*p* < 0.001), emotional forgiveness (*p* < 0.001), age (*p* = 0.002), and sex (*p* < 0.001). Fairness/cheating positively correlated with loyalty/betrayal (*p* < 0.001), authority/subversion (*p* < 0.001), sanctity/degradation (*p* < 0.001), decisional forgiveness (*p* < 0.001), emotional forgiveness (*p* = 0.033), age (*p* = 0.006), and sex (*p* < 0.001). Authority/subversion was positively linked with sanctity/degradation (*p* < 0.001), decisional forgiveness (*p* < 0.001), and emotional forgiveness (*p* < 0.001). Furthermore, sanctity/degradation exhibited positive associations with decisional forgiveness (*p* < 0.001) and emotional forgiveness (*p* < 0.001), while decisional forgiveness showed a positive relationship with emotional forgiveness (*p* < 0.001). Conversely, negative correlations emerged between loyalty/betrayal and sex (*p* = 0.002), as well as between authority/subversion and sex (*p* = 0.006). The comprehensive set of correlation coefficients is provided in Table 1. In a subsequent analysis, we explored the interaction between sex and age in relation to the studied variables. However, the findings indicated that these interactions were not statistically significant [*p*‐values (*p*s) > 0.05].

### 6.1 Latent Profile Analysis of Moral Foundations

**FIGURE 1:**
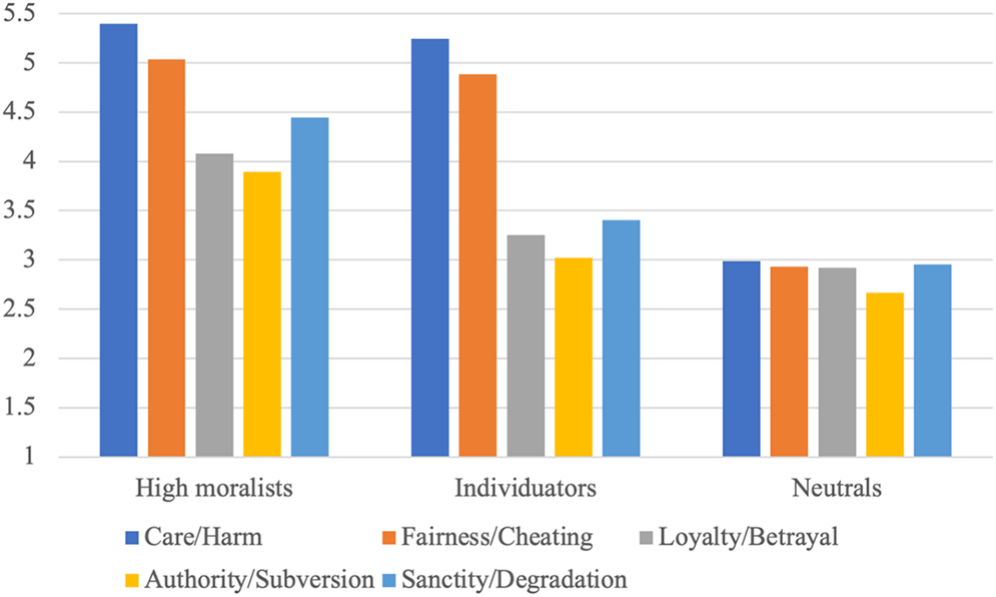
The three moral foundation profiles (*N* = 927).

Table 2 shows the statistical fit of the models and profile membership distribution concerning the moral foundations. The selected final model, which best fit the data based on the BIC, SSABIC, BLRT, and Entropy metrics, consisted of three distinct profiles. The average posterior probabilities were class 1 = 0.85, class 2 = 0.83, and class 3 = 0.76. This representation is illustrated in Figure 1, depicting the delineation of three distinctive moral foundation profiles in participants (*N* = 927). Graphs showing the different profiles (unconfirmed by data) are presented in Supplement 1.

**TABLE 2:** Fit statistics and profile membership distribution for the moral foundations (*N* = 927).

	Fit statistics				Profile membership distribution			
Model	BIC	SSABIC	BLRT	Entropy	1	2	3	4
One‐profile	10,589	10,557	N/A	N/A	927			
Two‐profile	9715	9664	−16871***	0.795	754	173		
Three‐profile	9270	9181	−16707***	0.821	185	476	266	
Four‐profile	9454	9384	–16476^ns^	0.815	223	537	145	22

*Note: ns* non‐significant.

*** *p* < 0.001.

The first class (*N* = 185) scored highly in each moral foundation, leading us to categorise them as the “high moralists”. In contrast, the second class (*N* = 476) displayed lower scores in care/harm and fairness/cheating compared to the former group, alongside moderate ratings in loyalty/betrayal, authority/subversion, and sanctity/degradation foundations. As a result of these discernible patterns, we classified this group as the “individuators”. The third class (*N* = 266) demonstrated the lowest scores across each moral foundation, prompting us to designate them as the “neutrals” (for means and standard errors, see Table 3).

**TABLE 3:** Means and standard errors of the three moral foundation profiles (*N* = 927).

	Class 1 (*N* = 185)		Class 2 (*N* = 476)		Class 3 (*N* = 266)	
	*M*	*SE*	*M*	*SE*	*M*	*SE*
Care/harm	5.40	0.03	5.24	0.04	2.99	0.07
Fairness/cheating	5.04	0.04	4.89	0.04	2.93	0.07
Loyalty/betrayal	4.08	0.06	3.25	0.06	2.92	0.07
Authority/subversion	3.89	0.08	3.02	0.06	2.67	0.08

### 6.2 Moral Foundation Differences in Forgiveness

The MANOVA revealed significant distinctions among the three profiles in terms of episodic forgiveness (encompassing both decisional and emotional forgiveness), evidenced by Pillai's trace (*F*_(4,1846)_ = 12.83, *p* < 0.001). Further univariate tests confirmed disparities across profiles in specific forgiveness domains: decisional forgiveness (*F*_(2,470)_ = 3.43, *p* = 0.033, *η* ^2^ = 0.03) and emotional forgiveness (*F*_(2,470)_ = 19.86, *p* < 0.001, *η* ^2^ = 0.22).

Posthoc assessments (employing Hochberg adjustment) showed significant differences in two of three posthoc comparisons for decisional forgiveness. Specifically, high moralists (*M* = 2.91, SD = 0.75), showed statistically significantly higher scores than individuators (*p* = 0.030; *M* = 2.75, SD = 0.72) and neutrals (*p* = 0.049; *M* = 2.76, SD = 0.74). Concerning emotional forgiveness, high moralists (*M* = 3.98, SD = 0.90) showed significantly higher scores than individuators (*p* < 0.001; *M* = 3.49, SD = 1.03) and neutrals (*p* < 0.001; *M* = 3.31, SD = 1.09). Additionally, individuators showed more emotional forgiveness than neutrals (*p* = 0.047).

## 7 Discussion

The current study examined the presence of moral foundations using the MFQ by Graham et al. (2013) and their correlation with the decisional and emotional dimensions of episodic forgiveness. As predicted, LPA identified discrete profiles based on MFQ scores, reflecting similar moral patterns found in studies by Milojev et al. (2014) and Greenway et al. (2019). However, our models best accommodated only three moral profiles (high moralists, individuators, and neutrals), unlike previous research identifying four classes, including an additional “moderates” group. It is noteworthy that earlier investigations were carried out in US and New Zealand populations, commonly characterised by minimal cultural differences from Canada (e.g. Bickford 2020). A potential explanation for the variance in the number of moral profiles could be that Canadians might demonstrate more polarised attitudes or a preference for more extreme ratings on the scales. However, this is only a conjecture and would require further investigation in future research to confirm. Despite a large sample in our study, further replications are imperative to definitively establish a consistent three‐profile moral framework within the Canadian population.

Moreover, the findings of this study align with the hypothesis proposed by Milojev et al. (2014), affirming the presence of diverse moral profiles that transcend the conventional individualising–binding binary framework. In contrast to the dichotomy postulated by Graham, Haidt, and Nosek (2009) of only two profiles (individualising vs. binding morals), our LPA conducted on the Canadian sample revealed three distinct profiles. The individuators profile corresponds to the typical pattern formerly associated with liberals—exhibiting heightened scores in care/harm and fairness/cheating and lower scores in loyalty/betrayal, authority/subversion, and sanctity/degradation, as Graham, Haidt, and Nosek (2009) identified. In contrast, the other two profiles resemble the conservative (binding) pattern observed by Graham, Haidt, and Nosek (2009), where all five moral foundations are rated more evenly; however, they differ in the strength of these ratings. Rather than displaying a consistent and uniform structure, our LPA results revealed two profiles demonstrating a similar configuration (i.e. relatively balanced ratings across the five moral foundations) but diverging in terms of rating intensity (i.e. high [high moralists] and low [neutrals] scores). Consequently, future research should consider a more nuanced delineation of moral profiles extending beyond the binary framework initially proposed by Graham, Haidt, and Nosek (2009).

Beyond that, MANOVA results indicated statistically significant differences in decisional and emotional forgiveness among the three identified profiles. Individuals categorised as high moralists demonstrated elevated scores in emotional and decisional forgiveness, while those classified as neutrals exhibited lower levels in both dimensions of episodic forgiveness. These observations align with the prevailing consensus in the literature, indicating that moral development significantly influences forgiveness (Bassett et al. 2018; Garcia 2011; Griswold 2007; Lindsey 2014). Interestingly, in the study conducted by Benard et al. (2022) investigating everyday intergroup conflicts within a US sample, forgiveness was regarded as more morally acceptable because it prevented chaos and averted larger conflicts that could result in unintended consequences compared to seeking revenge. Importantly, our research contributes to existing knowledge by highlighting that, within the two dimensions of episodic forgiveness, a preference for individualistic morality correlates exclusively with heightened levels of emotional forgiveness. Consequently, individuals prioritising moral considerations in harm/care and fairness/cheating may face greater difficulties in embracing decisional forgiveness. This observation is supported by the similarity in the low levels of decisional forgiveness observed among both the individuators and neutrals. Distinctive patterns in moral foundations could explain this phenomenon (Graham et al. 2011).

Given that this is, to our knowledge, the first study examining differences in episodic forgiveness with an exploratory nature, we cannot directly discuss our findings in the context of existing literature. However, insights from research on cultural orientation may help in understanding the observed effects. The moral foundations theory (Graham et al. 2013) posits that individualising moral foundations are prevalent in individualist cultural orientation, while binding moral foundations are more characteristic of collectivist cultural orientation. Despite this study being conducted in an individualistic country, high moralists exhibited a greater importance of moral values associated with a collectivist orientation than individuators, which potentially explains differences in forgiveness between these groups. Kadiangandu et al. (2007) proposed that in individualistic orientation, forgiveness is perceived as an internalised process focused on internal emotions, whereas in collectivistic orientation, it is viewed as an interpersonal act emphasising expressing forgiveness to the transgressor (decision‐making). Similarly, Hook, Worthington, and Utsey (2009) suggested that within individualistic orientation, emotional forgiveness holds greater significance than decisional forgiveness, stemming from individualistic individuals prioritising personal peace over their behaviourtowards the offender. Additionally, Ho and Fung (2011) theorised that individualists seek differentiation and personal gains, potentially aligning more with emotional forgiveness. In contrast, collectivists prioritise collective norms, social harmony, and relationships, potentially favouring decisional forgiveness. However, it is important to note that while the moral foundations theory intersects with the cultural dimension of individualism vs. collectivism, our findings should not be generalised to the cultural impact of forgiveness. The observed trends specifically relate to moral evaluations.

The literature primarily links forgiveness with addressing care/harm and fairness/cheating (Enright, Freedman, and Rique 1998; Worthington 2020), suggesting a narrow focus on these two moral concerns. Extending Lindsey's (2014) research, our study challenges this limited perspective, revealing that individuals consider five distinct dimensions when evaluating the moral justifiability of forgiveness. This expanded viewpoint is evident not only in observed differences among the three moral profiles but also in statistically significant, albeit marginal, positive correlations between ratings within each moral foundation and the intensity of decisional and emotional forgiveness.

These findings bear theoretical implications. It is essential to highlight the definition of forgiveness from the *APA Dictionary of Psychology* as “willingly setting aside feelings of resentment toward an individual who has committed a wrongdoing, acted unfairly, or caused harm in some manner.”. (VandenBos 2007, 385), emphasising harm and cheating as necessitating forgiveness. However, our research indicates the necessity of broadening the definition of forgiveness to encompass transgressions related to additional moral concerns, such as loyalty/betrayal, authority/subversion, and sanctity/degradation.

In our study, significant disparities in moral foundations were observed between the sexes. Compared to males, females tended to prioritise care/harm and fairness/cheating in their behavioural decisions and exhibited lower tendencies towards loyalty/betrayal and authority/subversion foundations. These findings align with the observations made by Niazi, Inam, and Akhtar (2020) and Skalski‐Bednarz et al. (2023), which emphasise women's consistent inclination towards individualising‐oriented perspectives and men's greater consistency in binding‐oriented inclinations. These distinctions in moral foundations contribute to a more comprehensive understanding of how sex and gender may influence moral reasoning.

The practical implications of our findings regarding the relationship between moral foundations and episodic (decisional and emotional) forgiveness are significant. Understanding these connections can facilitate tailored forgiveness interventions aligned with individuals' moral profiles, addressing specific challenges related to forgiveness based on diverse moral tendencies. For example, people emphasisingindividualising foundations might benefit from interventions focusing on enhancing loyalty, authority, and sanctity as motivators to improve their lower levels of decisional forgiveness. Furthermore, those with a lower overall emphasis on moral values might also find supplementary value in strategies emphasising care and fairness, enhancing both emotional and decisional forgiveness. Additionally, recognising the influence of moral foundations on forgiveness can enrich conflict resolution approaches by addressing inherent moral concerns and aligning solutions with individuals' unique moral orientations. These insights are crucial for crafting precise interventions targeting forgiveness and conflict resolution, ultimately leveraging and respecting the diversity of moral orientations. An example of an existing form of training, which could be currently applicable just before conventional forgiveness education (see e.g. Worthington 2019), is a mindfulness intervention aimed at enhancing reflective awareness and a controlled sense of self in the present moment. In recent research, Verhaeghen and Aikman (2020) demonstrated that this intervention could increase the relevance of binding aspects of morality through changes in self‐transcendence. Additionally, reflective awareness, developed in the mindfulness program, is associated with individualising moral aspects.

While employing LPA has enriched our understanding of the connections between moral foundations and episodic forgiveness, our study has limitations. The study does not explore potential contextual or situational influences that might moderate the identified relationships. Future research should consider integrating such factors to comprehensively examine the nuanced interplay between moral foundations and forgiveness dynamics. Moreover, it is crucial to acknowledge that our sample consisted of the Canadian population, characterised by an individualistic culture. As such, caution should be exercised when extending our findings to populations with different cultural orientations, particularly collectivist cultures. To enhance the external validity and broaden the applicability of our conclusions, future studies should intentionally include diverse cultural samples, allowing for a more nuanced understanding of how cultural contexts shape moral orientations and influence responses to forgiveness. In addition, a notable limitation of our study is the absence of control over the specific type or nature of transgressions for which forgiveness was assessed in participants. The nature of the transgressions may influence the variability in responses to forgiveness, and future research should consider incorporating controlled transgression types to provide a clearer picture of the relationship between moral foundations and forgiveness.

## 8 Conclusions

The moral foundations theory emerged as a synthesis of diverse streams of research in moral cognition from social and biological sciences (Graham et al. 2013). In this study, we expand on this framework, utilising the moral foundations theory to delineate broader moral profiles and pioneering exploration into the relationship between these moral foundations and episodic forgiveness. Our analysis of MFQ scores revealed three distinct moral foundation profiles, each exhibiting varying associations with the decisional and emotional dimensions of episodic forgiveness. Notably, the high moralist group displayed higher scores in both forgiveness dimensions than individuals in the individuator and neutral groups. Conversely, those in the individuator group reported elevated emotional forgiveness compared to the neutrals. These findings underscore the utility of moral profiling based on the moral foundations theory in predicting episodic forgiveness, opening avenues for further inquiry into the intersection of morality and forgiveness.

## Ethics Statement

All procedures performed in studies involving human participants were in accordance with the ethical standards of the institutional and/or national research committee and with the 1964 Helsinki Declaration and its later amendments or comparable ethical standards. This study was approved by the Ethics Committee of the University of Economics and Human Sciences in Warsaw (Research project approval # 5/III/2023). Informed consent was obtained from all individual participants included in the study.

## Conflicts of Interest

The authors declare no conflicts of interest.

## Supporting information


**Data S1. Supplement 1.** Alternative Models (Unconfirmed by Data).
**Figure S1.** Single‐Class Moral Foundation Profile (Unconfirmed by Data).
**Figure S2.** Two‐Class Moral Foundation Profiles (Unconfirmed by Data).
**Figure S3.** Four‐Class Moral Foundation Profiles (Unconfirmed by Data).

## Data Availability

The data that support the findings of this study are available on request from the corresponding author, Sebastian Binyamin Skalski‐Bednarz. The data are not publicly available due to restrictions (their containing information that could compromise the privacy of research participants).
