# Supplementary material for: Latent Profile Analysis of Moral Foundations: Emotional and Decisional Forgiveness Approaches to Models of Morality
Source: Int J Psychol. 2025 Feb 3;60(2):e70009. doi: 10.1002/ijop.70009 (PMC11799746; doi:10.1002/ijop.70009)
Supplement: Supplementary file 1 — Data S1. Supplement 1. Alternative Models (Unconfirmed by Data). Figure S1. Single‐Class Moral Foundation Profile (Unconfirmed by Data). Figure S2. Two‐Class Moral Foundation Profiles (Unconfirmed by Data). Figure S3. Four‐Class Moral Foundation Profiles (Unconfirmed by Data). [file IJOP-60-e70009-s001.docx]

**Supplement 1**

*Alternative Models (Unconfirmed by Data)*

**Figure 1**

*Single-Class Moral Foundation Profile (Unconfirmed by Data)*

**Figure 2**

*Two-Class Moral Foundation Profiles (Unconfirmed by Data)*

**Figure 3**

*Four-Class Moral Foundation Profiles (Unconfirmed by Data)*
